# Postural Stability of Patients with Schizophrenia during Challenging Sensory Conditions: Implication of Sensory Integration for Postural Control

**DOI:** 10.1371/journal.pone.0158219

**Published:** 2016-06-29

**Authors:** Ya-Ling Teng, Chiung-Ling Chen, Shu-Zon Lou, Wei-Tsan Wang, Jui-Yen Wu, Hui-Ing Ma, Vincent Chin-Hung Chen

**Affiliations:** 1 Institute of Allied Health Sciences, College of Medicine, National Cheng Kung University, Tainan, Taiwan; 2 Department of Occupational Therapy, College of Medical Science and Technology, Chung Shan Medical University, Taichung, Taiwan; 3 Occupational Therapy Room, Chung Shan Medical University Hospital, Taichung, Taiwan; 4 Department of Psychiatry, Cen-Der Hospital, Taichung, Taiwan; 5 Department of Psychiatry, Chung Shan Medical University Hospital, Taichung, Taiwan; 6 Department of Occupational Therapy, College of Medicine, National Cheng Kung University, Tainan, Taiwan; 7 Department of Psychiatry, Chiayi Chang Gung Memorial Hospital & Chang Gung University, Chiayi, Taiwan; 8 School of Medicine, Chang Gung University, Taoyuan, Taiwan; Ludwig-Maximilian University, GERMANY

## Abstract

Postural dysfunctions are prevalent in patients with schizophrenia and affect their daily life and ability to work. In addition, sensory functions and sensory integration that are crucial for postural control are also compromised. This study intended to examine how patients with schizophrenia coordinate multiple sensory systems to maintain postural stability in dynamic sensory conditions. Twenty-nine patients with schizophrenia and 32 control subjects were recruited. Postural stability of the participants was examined in six sensory conditions of different level of congruency of multiple sensory information, which was based on combinations of correct, removed, or conflicting sensory inputs from visual, somatosensory, and vestibular systems. The excursion of the center of pressure was measured by posturography. Equilibrium scores were derived to indicate the range of anterior-posterior (AP) postural sway, and sensory ratios were calculated to explore ability to use sensory information to maintain balance. The overall AP postural sway was significantly larger for patients with schizophrenia compared to the controls [patients (69.62±8.99); controls (76.53±7.47); *t*_1,59_ = -3.28, *p<*0.001]. The results of mixed-model ANOVAs showed a significant interaction between the group and sensory conditions [*F*_5,295_ = 5.55, *p<*0.001]. Further analysis indicated that AP postural sway was significantly larger for patients compared to the controls in conditions containing unreliable somatosensory information either with visual deprivation or with conflicting visual information. Sensory ratios were not significantly different between groups, although small and non-significant difference in inefficiency to utilize vestibular information was also noted. No significant correlations were found between postural stability and clinical characteristics. To sum up, patients with schizophrenia showed increased postural sway and a higher rate of falls during challenging sensory conditions, which was independent of clinical characteristics. Patients further demonstrated similar pattern and level of utilizing sensory information to maintain balance compared to the controls.

## Introduction

Postural dysfunctions have been reported in patients with schizophrenia [1–4]. Studies that assessed balance using the tandem gait or the Romberg test administered during neurological examinations indicated significantly poor standing balance in patients with schizophrenia compared to that of healthy subjects [3, 5, 6]. In addition, image studies showed a change in the volume of cerebellum in schizophrenia [7–9], which is associated with compromised sensory integration [10] and poor postural sequences [1,11, 12]. Both cerebellar etiologies as well as balance dysfunctions were found even in antipsychotic naïve patients in early stages of illness [8, 9], suggesting that postural control dysfunctions are part of the intrinsic nature of schizophrenia. However, balance performance had been examined by rater-dependent observations in most studies and rendered the objectivity questionable [6]. Moreover, these studies focused mainly on overall functioning; therefore, the mechanisms underlying postural dysfunctions in schizophrenia remain largely unclear [13]. Recently, a few studies have used pressure sensitive posturography to assess postural control of patients with psychotics and reported significant increase in sway area and total COP excursion length compared to that of healthy controls [1, 4, 14–16]. This indicates increased postural sways of psychotic patients in quiet standing and provides more objective information on postural control of patients with schizophrenia.

Normal postural control is characterized by the integration of sensory information from somatosensory, visual, and vestibular systems to provide body frames of reference in space [17, 18]. However, these sensory mechanisms have been reported to be compromised in patients with schizophrenia [19–25], as well as sensory integrative dysfunction [1] as demonstrated by significantly lower scores on the sensory integration subscale of the Neurological Examination Scale [9]. These abnormal sensory consequences may be associated with difficulties with on-line corrections of movements [26], and they may compromise abilities to adjust the center of gravity within the base of support to maintain balance. Studies have reported significantly increased postural sway in conditions with visual deprivation compared to eyes open conditions in patients with schizophrenia [1, 16]. However, some studies have indicated that the presence of visual information did not enhance standing balance in patients with schizophrenia, as it did in normal subjects [4, 14, 27]. Sullivan et al. [15] even reported greater postural sway in eyes open condition compared to that in eyes closed condition and suggested impaired visuomotor integrative abilities in patients with schizophrenia. Other studies examining somatosensory influence on postural control also reported increased postural sway compared to controls in conditions where participants standing with both feet close together [16] or standing on a yielding surface [28]. Postural stability was further compromised when the visual input was removed [16]. Kent and coworkers [1] also reported that the complexity of postural sway of patients with schizophrenia did not decrease upon withdrawal of visual input as the controls did, suggesting problems with integrating higher-frequency feedbacks, including somatosensory information for postural control.

Balance studies have not consistently explored the relationships between standing balance and clinical characteristics in patients with schizophrenia, and controversial results have been reported. Some studies reported correlations between postural stability and either general psychiatric symptoms [1] or negative psychiatric symptoms [28]. Similar studies have suggested that the use of quetiapine [2] and risperidone [13] increases postural sway. However, other studies have failed to detect associations with psychiatric symptoms [15] or relationships with chlorpromazine equivalent dose (CPZE) of antipsychotics [1, 2, 4], medication type (typical versus atypical) [15], the use of benzodiazepines [2], or dose of risperidone [13]. Regarding the extrapyramidal symptoms prevalent in patients with schizophrenia [29, 30], only one study has examined relationships between standing balance and clinical characteristics and reported no correlations with postural stability [4].

In summary, postural control in schizophrenia has been surprisingly understudied [1, 3, 14]. Despite of crucial roles that multiple sensory systems play in postural control, few studies have investigated how patients with schizophrenia organize visual, vestibular, and somatosensory information to maintain postural control. The existing studies on the relationships of clinical characteristics with standing balance provided controversial findings. Therefore, the purpose of this study was to investigate postural stability of patients with schizophrenia during challenging sensory conditions. Most previous studies have examined postural stability of patients with schizophrenia in static environments. In this study, dynamic sensory conditions that interfere with normal postural responses were introduced to test postural stability. By comparing balance performance across different sensory conditions, sensory integration was explored to investigate how patients with schizophrenia coordinate multiple sensory systems to maintain standing balance. In addition, the relationships between postural stability and clinical characteristics were also examined. The hypotheses were that compared to healthy subjects, patients with schizophrenia would demonstrate poor postural stability and use sensory information differently in dynamic contexts with challenging sensory information.

## Materials and Methods

### Participants

Twenty-nine patients with schizophrenia were recruited from the day care at the department of psychiatry in Chung Shan Medical University Hospital. The inclusion criteria were patients diagnosed with schizophrenia by senior psychiatrists according to DSM-IV criteria. The participants were symptomatically stable, and had stable use of medication two weeks prior to the testing. Patients with (1) musculoskeletal, neurological and vestibular diagnosis, (2) visual impairments that could not be corrected by optic lenses, (3) history of alcoholism or substance abuse, and (4) those with cognitive impairments as verified by scores lower than 24 on Mini-Mental State Examination (total score 30) were excluded. Thirty-two healthy adults matched to the patient group for age, gender, height, and weight were recruited from hospital staffs and families of patients who received services in the Physical Medicine and Rehabilitation Department in the same hospital to serve as the controls. In addition to the same exclusion criteria applied to the patient group, the primary investigator conducted interviews to exclude those with present and past psychotic disorders, as well as those with family history of psychosis.

Table 1 shows the participants’ demographic and clinical characteristics. The mean age of patients with schizophrenia was 37.59±8.6 years old, with the mean illness duration of 13.72±6.92 years. All patients with schizophrenia received atypical antipsychotics and attended the work rehabilitation program daily in the hospital. Their work activities included delivering charts, re-shelving books in the library, answering phone calls, providing information at the information desk, and keying the data, among others. No significant differences were found between patients with schizophrenia and the healthy controls in terms of age, gender, height, weight, and cognitive function, as measured by the MMSE scores.

**Table 1:** Demographic data of participants.

Variable/group	Schizophrenia	Controls	*p*
Number of participants	29	32	
Gender (male/female)	11/18	10 /22	.60
Age (years)	37.59±8.60	37.63±8.87	.80
Height (cm)	162.36±6.82	163.08±8.82	.06
Weight (kg)	73.19±14.90	74.14±14.92	.62
MMSE ^a^ score	29.31±1.37	29.47±1.08	.48
Illness duration (yrs)	13.72±6.92	N/A	
BPRS^b^	9.17±4.94	N/A	
AIMS^c^	3.34±3.43	N/A	
Levels of antipsychotics (CPZE^d^, mg)	377.43±260.36	N/A	
Benzodiazepines (yes/no)	11/18	N/A	
Antidepressants (yes/no)	7/22	N/A	

^a^MMSE: Mini-mentalState Examination

^b^BPRS: Brief Psychiatric Rating Scale

^c^AIMS: Assessment of Involuntary Movement

^d^CPZE: Chlorpromazine equivalent dose

### Ethics Statement

**Fig 1:**
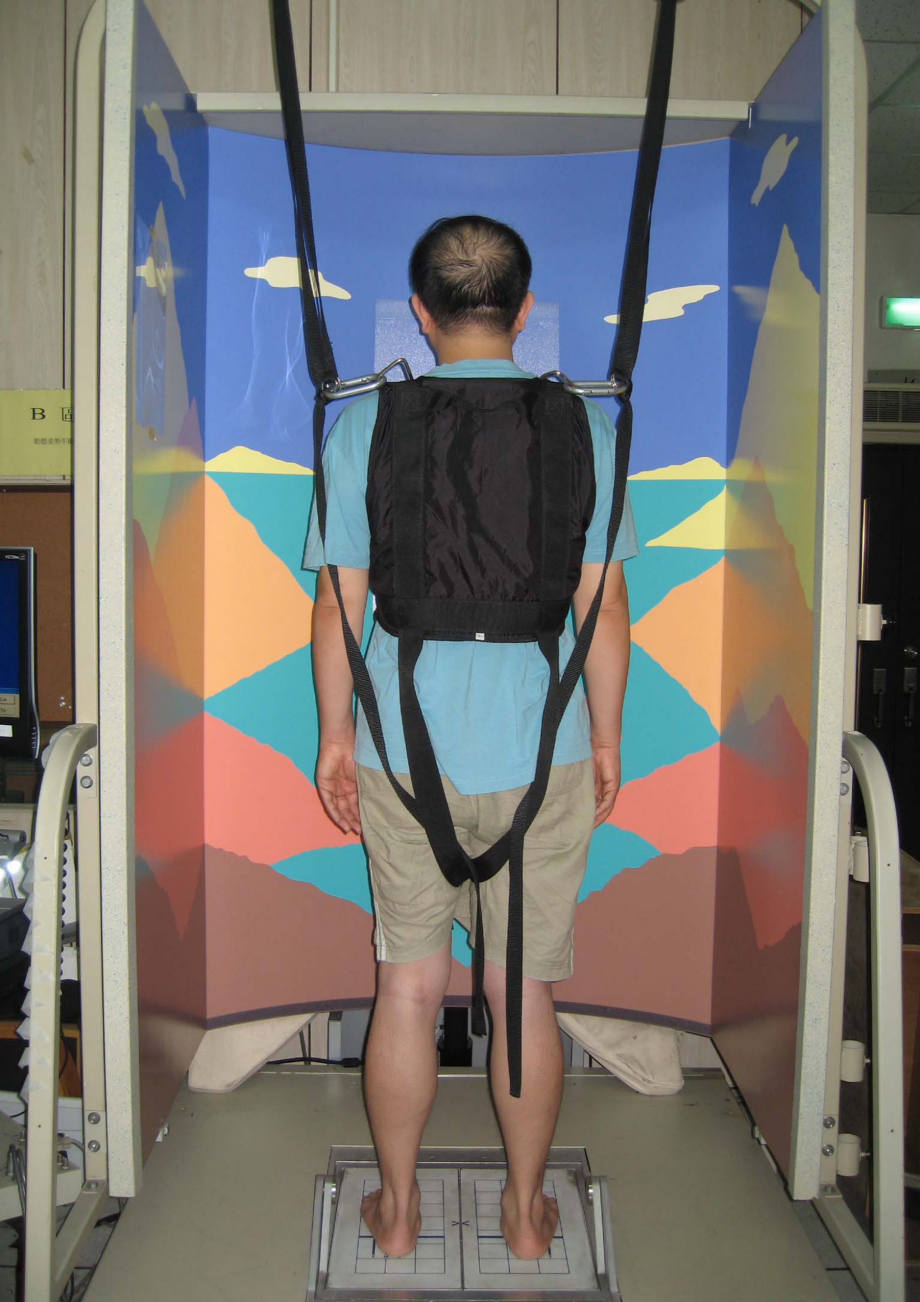
Smart balance master.

The Institution Review Board of Chung Shan Medical University Hospital approved the study. All schizophrenic participants were legally competent, and no legally authorized representative consented on behalf of participants. A senior psychiatrist assessed the capacity of the patients to understand and provide informed consent. Prior to the study, the study procedure and risks were explained. Subsequently, all subjects provided written informed consents in accordance with the Declaration of Helsinki. Furthermore, a nurse staff of the day care acted as the witness during the informed consent process. The psychiatrist and the nurse were not in the research team of this study. Additionally, the participant appeared in Fig 1 in this manuscript had given written informed consent (as outlined in PLOS consent form) to publish the details of this case.

### Measures

#### Clinical measurements

Psychiatric symptoms of patients with schizophrenia were measured by the Brief Psychiatric Rating Scale (BPRS) [31]. Scale items were measured on a 6-point ordinal scale ranging from 0 indicating no symptom to 5 indicating the most severe symptom. The severity of negative psychiatric symptoms was evaluated by the BPRS negative symptom subscale. In addition, movement disorders were measured by the Abnormal Involuntary Movement Scale (AIMS) [32], which evaluated abnormal movements over the facial, oral, extremity, and truncal areas. Three other items, including the overall severity of abnormal movements, incapacitation caused by abnormal movements, and self-awareness of abnormal movements, were also examined and measured on a scale from 0 indicating no symptom to 4 indicating the most severe symptoms.

#### Postural control measurement

Postural control data were recorded by the Smart Balance Master (NeuroCom Inc., Clackamas, OR, USA), which was equipped with a movable visual surround and two computerized force plates mounted on the support surface for standing (Fig 1). The forceplates allowed for toes-up and down movements around ankle joints and also captured changes in the center of pressure, from which inferences of the location of the center of gravity can be made. The sampling rate of the force plates was 100Hz. A suspension harness was loosely provided to prevent the participants from contact with the floor should they lose balance during testing. A computer monitor was mounted in the front wall to provide instant feedback of body sway.

### Procedure

**Fig 2:**
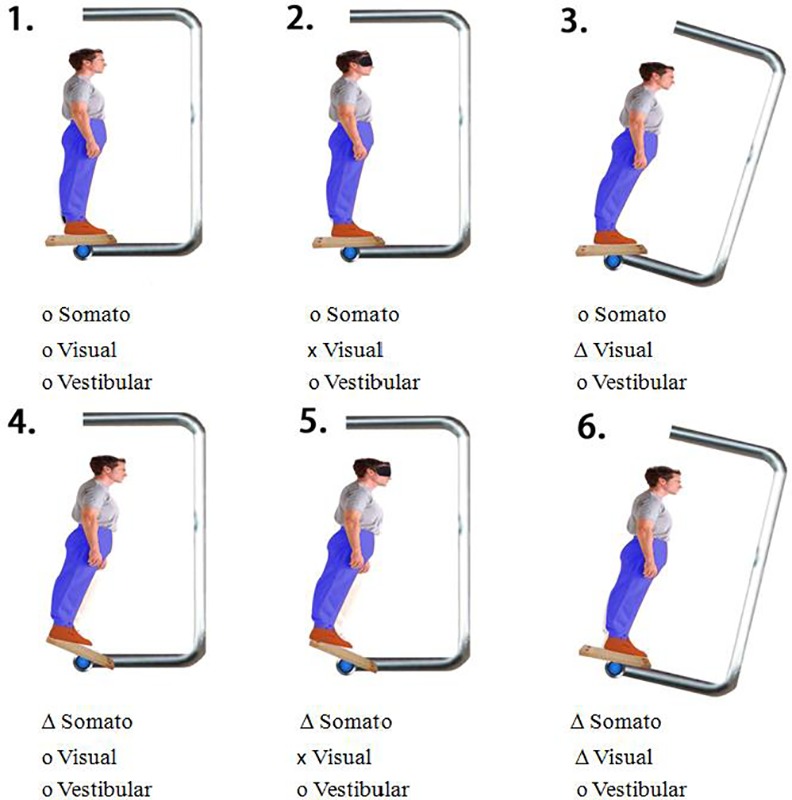
Six sensory conditions. o: Correct; x: Removed; **△**:Misleading sensory input.

A counterbalanced repeated-measure design was used. The sensory organization test [33] was adopted to test postural stability of the participants. All participants stood barefoot on the support surface with designated feet positions based on their body height, as described in the manual, and they placed their arms along trunk sides while looking at the monitor. The participants were asked to maintain the upright position without moving their feet. They experienced six sensory conditions that varied in level of congruency of somatosensory, visual, and vestibular information by selectively disrupting somatosensory and/or visual input. The six sensory conditions were (1) eyes open with both fixed support surface and visual surround, (2) eyes closed with both fixed support surface and visual surround, (3) eyes open with fixed support surface and sway-referenced visual surround, (4) eyes open with sway-referenced support surface and fixed visual surround, (5) eyes closed and sway-referenced support surface and fixed visual surround, and (6) eyes open with both sway-referenced support surface and visual surround (see Fig 2 for sensory information of each condition). In sway-referenced conditions (i.e., condition 3 through condition 6), the sway gain was set at 1.0 so that either the support surface or the visual surround or both moved in the same direction with the same amount of amplitudes and speed as the subject swayed. The faster the participant leaned forward or backward, the faster the device moved. Sway referencing minimizes ankle movements or self motion with respect to the visual scene that typically accompanies postural sway. For example, when the participants swayed forward, the support surface produced a toes-down tilt and the visual surround a forward rotation, thereby minimizing the somatosensory and visual input. This provided misleading somatosensory or/and visual information in relation to the orientation of the body in space that was incongruent with other sensory information [34]. Accordingly, it produced instantaneous challenges for the participants to reweigh sensory information and maintain balance. The more the subjects swayed in dependence on the sensory contexts changes, the greater the dynamic challenge to maintain balance. Each condition was repeated in three trials, with each trial lasting for 20 seconds. Both the sequence and the order of testing conditions were counterbalanced, and the subjects were allowed to rest between trials.

### Postural Variables

#### Equilibrium scores

Trajectories of the COP for each trial were recorded to estimate the center of gravity (COG) using the inverse dynamics approach [34, 35]. The anterior-posterior (AP) COG sway angle (*θ*) was measured by the angle between the vertical line extended from the center of the foot support and the line projecting from the center of the foot support to the COG [36]. The equilibrium scores were subsequently derived to illustrate how well anterior-posterior (AP) postural sways of the participants remained within the AP sway angular range of clinically healthy adults. The formula is as follows:

$$\text{Equilibrium score}=\frac{12.5{}^{\circ}-(\theta ant-\theta post)}{12.5{}^{\circ}}\times 100\%$$

Where *θ ant* is the maximum anterior sway angle, *θ post* is the maximum posterior sway angle in the same trial, and 12.5 degrees is the average body angular range for the AP postural sway of healthy adults in static standing [36]. A score of 100% indicates no body movement at all during static standing while 0% indicates a postural sway beyond the normal range or a fall. Stepping with either foot away from the initial position was also deemed a fall. Both the mean equilibrium score for each condition and a composite equilibrium score across six conditions were obtained. The mean equilibrium score was the average of the equilibrium scores for the three trials of a single condition. The composite equilibrium score was calculated using the following formula [34]:

$$\text{Composite equilibrium score}=\frac{ES1+ES2+3(ES3+ES4+ES5+ES6)}{14}$$

where *ES*1 through *ES*6 are the mean equilibrium scores for conditions 1 through 6, respectively. For the composite equilibrium score, more weight is given to the equilibrium scores for conditions 3 through 6 to account for their increased challenges to postural control.

#### Sensory ratios

By comparing the mean equilibrium scores (ES) for specific conditions, we calculated sensory ratios for sensory analysis to determine the contribution of sensory systems to maintain standing balance [37, 38]:

$$\text{Somatosensory ratio}=\frac{ES2}{ES1};\;\text{Visual ratio}=\frac{ES4}{ES1};\;\text{Vestibular ration}=\frac{ES5}{ES1}$$

The somatosensory ratio indicates the ability to use somatosensory information, which is the most dominant sensory system for postural control among the three sensory systems, by calculating the extent of stability loss when the visual input is eliminated. Although the vestibular input may be the second complementary information to the visual input, it is less sensitive compared to the somatosensory input [33]. Similarly, the visual ratio indicates the ability to use visual information for controlling postural stability when correct somatosensory input is not available; and the vestibular ratio indicates the ability to rely on vestibular information exclusively for maintaining postural stability when correct somatosensory and visual inputs are not available. A ratio close to 1 indicates a superior ability to use that specific sensory information to maintain balance [35]. In addition, a visual preference ratio $\frac{ES3+ES6}{ES2+ES5}$ was calculated to inspect the extent of over-reliance of visual information even when it did not provide correct information regarding body orientation in space. Since visual information was unreliable in sway-referenced visual surround conditions, similar to that in eyes closed conditions [35], it was neglected by the participants, resulting in a visual preference ratio close to 1. A value less than 1 suggests a tendency of over-reliance on visual information.

### Statistical Analysis

Statistical analysis was performed using SPSS, version 18.0 (SPSS Inc, Chicago, IL, USA). Independent *t*-tests and chi-square tests were used to test group differences in demographic data, the frequency of falls, the composite equilibrium scores, and sensory ratios between the two groups. The effects of sensory conditions on the equilibrium scores were further examined using mixed-model analysis of variances (ANOVAs) with group being the between-subject factor and sensory conditions as the within-subject factor. Post hoc tests were carried out using the least significance difference (LSD) test. In addition, Pearson correlations were conducted to examine the relationships among clinical characteristics, including the illness duration, scores on BPRS, negative BPRS, AIMS, CPZE of antipsychotics, and the composite equilibrium score as well as mean equilibrium scores for each sensory condition in patients with schizophrenia. The α level was set at 0.01.

## Results

### Postural Stability

**Fig 3:**
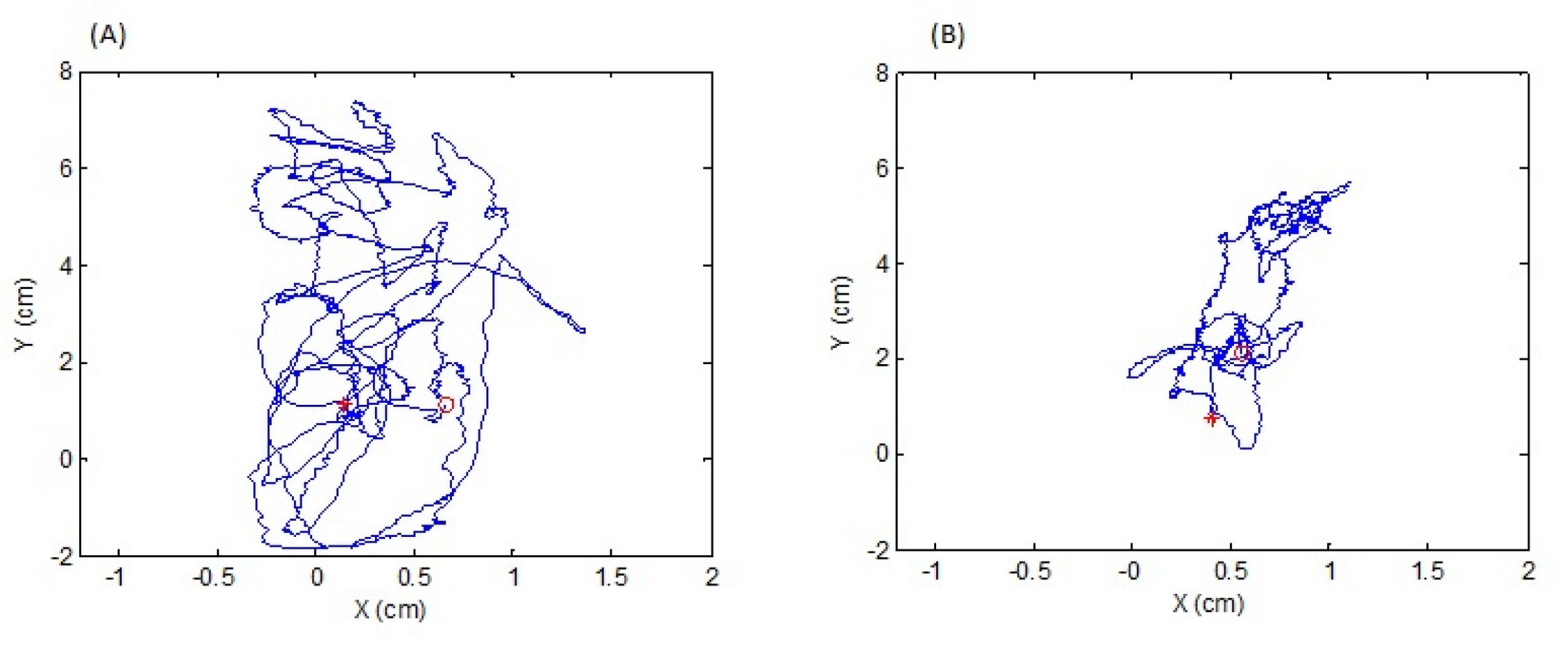
Excursion of the center of pressure (COP). Excursion of COP in condition 6 (A) for a patient with schizophrenia (B) for a control participant. Y-axis indicates anterior-posterior movements; X-axis indicates right-left movements.

Upon visual inspection of the excursion of the center of pressure (COP), the patients with schizophrenia showed more postural sway compared to the controls in the sensory organization test. Fig 3 exemplifies the excursion of the COP for one patient and one control subject in condition 6. For both groups, most falls occurred in condition 5 and 6, with schizophrenia patients falling significantly more compared to the controls [χ^2^_1,61_ = 7.11, *p<*0.01]. Throughout the testing, the patients were two times more likely to fall compared to the controls (28 falls for the patients, 13 falls for the controls).

**Fig 4:**
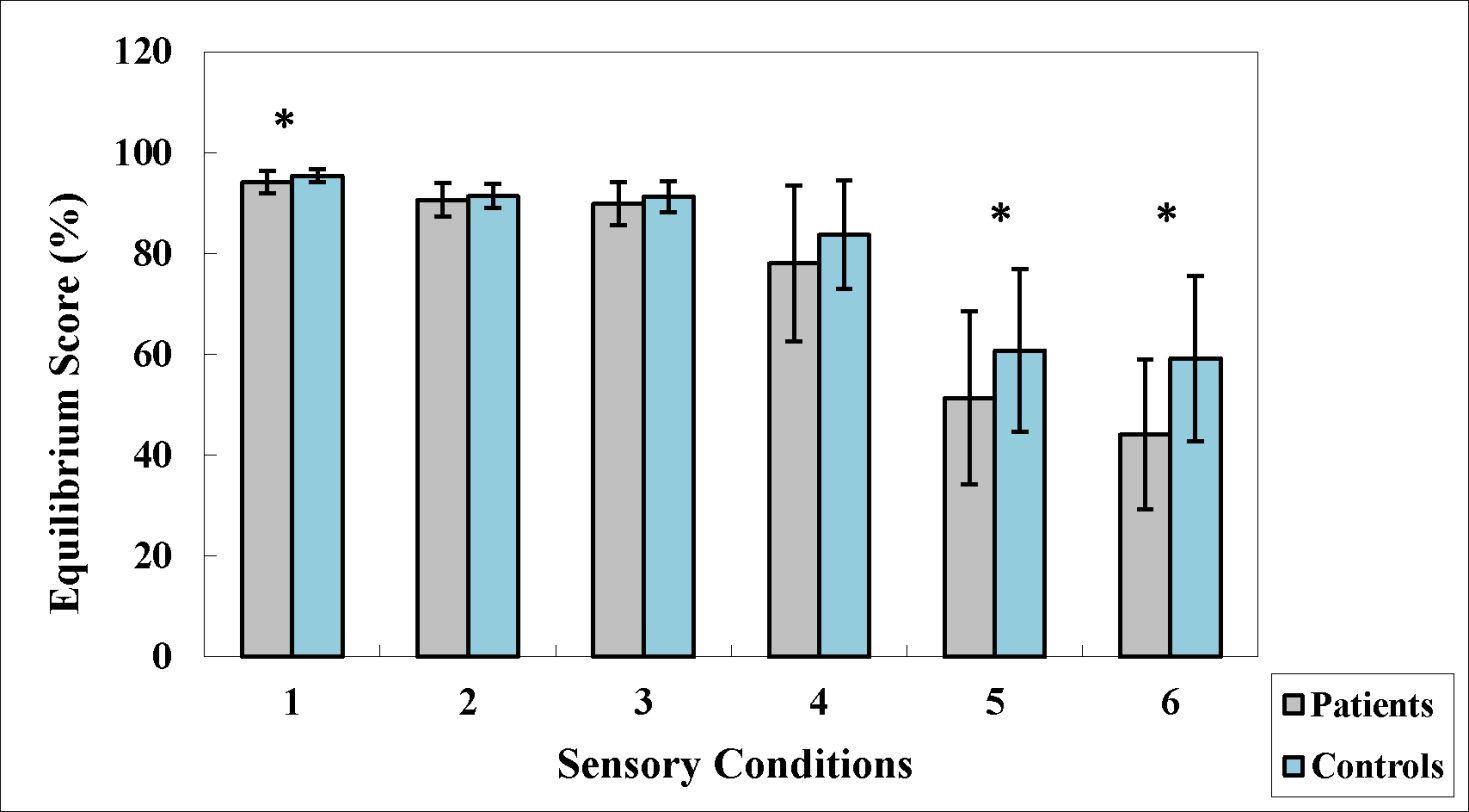
Mean Equilibrium scores for each sensory condition.

The mean equilibrium scores decreased subsequently from condition 1 to condition 6, and this pattern was observed for both groups. The results of the *t* test showed that patients with schizophrenia had a significantly lower composite equilibrium score compared to the control group [patients (69.62±8.99); controls (76.53±7.47); *t*_1,59_ = -3.28, *p<*0.001], indicating that patients with schizophrenia demonstrated significantly greater AP postural sway overall. The results of mixed-model ANOVAs showed a significant group × condition effect [*F*_5,295_ = 5.55, *p<*0.001], a significant group effect [*F*_1,59_ = 10.92, *p<*0.001], and also a significant condition effect [*F*_5, 295_ = 244.64, *p<*0.001]. Post hoc tests revealed that the mean equilibrium scores were significantly lower for patients with schizophrenia compared to the controls in condition 1, condition 5, and condition 6 (Fig 4). In addition, the results of post hoc tests also showed significant differences in the mean equilibrium scores for almost all pairwise comparisons between two conditions for both groups. Specifically, scores for the conditions with subsequent numbers were significantly lower compared to those for the conditions with preceding numbers. However, few exceptions emerged. Overall, no differences emerged between the mean equilibrium scores for conditions 2 and 3 and those for conditions 5 and 6 in the control group or between the mean equilibrium scores for conditions 2 and 3 in patients with schizophrenia.

#### Sensory Analysis

For both groups, the sensory ratios decreased from the somatosensory ratio [patients (0.96±0.03); controls (0.96±0.02)], to the visual ratio [patients (0.83±0.16); controls (0.87±0.11)], and the vestibular ratio [patients (0.54±0.18); controls (0.64±0.17)]. All sensory ratios for patients with schizophrenia, including visual preference ratio [patients (0.94±0.11); controls (0.99±0.12)], were lower than the ratios for the controls except that the somatosensory ratios were equivalent for both groups. No significant differences were found between patients with schizophrenia and controls in somatosensory ratio [*t*_1,59_ = 0.78, *p* = 0.44], visual ratio [*t*_1,59_ = -1.42, *p* = 0.16], or visual preference [*t*_1,59_ = -1.53, *p* = 0.13]. Although the vestibular ratio for patients with schizophrenia did not reach the statistical significance, a marginal value [*t*_1,59_ = -2.46, *p* = 0.02] suggested that patients had slightly lower vestibular ratio.

### Correlations with Clinical Characteristics

The results of Pearson correlation tests showed no significant correlations among postural variables, including the composite equilibrium score and the mean equilibrium scores, and clinical characteristics, which were the duration of illness, scores for BPRS, scores for BPRS negative symptom subscale, AIMS scores, and chlorpromazine equivalent dose of antipsychotics (all *p* values > 0.01).

## Discussion and Conclusions

To our knowledge, this is the first study to investigate postural stability of patients with schizophrenia in conflicting sensory environments by disrupting somatosensory and visual inputs selectively to create incongruence between sensory information. Instead of using conventional static conditions for balance examination, dynamic sensory contexts were provided to produce instantaneous postural challenges, and the ability to integrate sensory information and choose functionally appropriate input to maintain balance was explored.

### Postural Stability

In support of our hypotheses and previous findings [1, 2, 6], the current results demonstrated that patients with schizophrenia have significantly more difficulties maintaining standing balance, that they fall more and have significantly greater postural sway in AP direction overall compared to that of the controls. Further analyses indicated that schizophrenia patients swayed significantly more compared to the controls in baseline condition with all three sensory inputs provided correctly (condition 1) as well as in the conditions that provided unreliable somatosensory input either without visual input (condition 5) or with unreliable visual information (condition 6). However, although a significant difference between the two groups was found in condition 1, the absolute difference was small and the significance might be accounted for by small within group differences for both groups. Thus, it should not be viewed as clinically meaningful. Therefore, it can be concluded that the two groups performed similarly at baseline condition. This result differs from those reported in previous studies, which found significantly greater postural sway in patients with schizophrenia compared to controls when standing quietly on a fixed support surface [4, 14–16, 28]. Since our testing trials lasted for a rather brief time of 20 seconds, which is short compared to 30 to 180 seconds in similar static conditions reported in studies [39], therefore, the test presented minor postural challenges to our participants in baseline condition, including patients with schizophrenia, and might not be able to discriminate between those with good postural stability and those with moderate postural stability.

### Sensory Integration and Utilization of Sensory Information

For healthy adults, during transient perturbations to balance when standing on fixed support surfaces, somatosensory input provides the most unbiased information of body frames of reference in space compared to visual and vestibular systems [17]. It also elicits postural muscle responses with the shortest latency, followed by visual and then vestibular input [17, 33]. In our study, similar to the controls, patients with schizophrenia demonstrated a preference for somatosensory rather than visual and vestibular input. The value for the somatosensory ratio was the largest, followed by that for the visual ratio and finally the vestibular ratio. When the sensory ratios were compared between patients with schizophrenia and the controls, the patients demonstrated comparable ability to use somatosensory and visual information to maintain standing balance, and they managed to maintain postural stability at a level similar to that of the controls in the first four conditions. However, when visual input was unavailable (condition 5) or incongruent with vestibular information (condition 6) in conditions with unreliable somatosensory information, patients with schizophrenia demonstrated difficulties relying on vestibular information alone for balance control. As a result, they displayed significantly increased postural sway compared to controls and even fell. A marginal value of low vestibular ratio suggested that patients have trouble utilizing vestibular information to achieve postural control.

In addition, the controls demonstrated equivalent levels of postural stability in both conditions 2 and 3 as well as in conditions 5 and 6 with a visual preference ratio approaching 1, suggesting that they were able to ignore misleading visual information in sway-referenced surround conditions, similar to that in conditions with no visual input [33]. On the contrary, patients with schizophrenia showed significantly greater postural sway in condition 6 than in condition 5, indicating an inappropriate preference for visual information in the absence of reliable somatosensory information, regardless of the accuracy of visual input. Nevertheless, the differences between patients and controls in visual preference did not reach statistical significance.

Andreasen and coworkers proposed a distributed parallel network model to account for diverse symptoms observed in patients with schizophrenia [40, 41]. According to their cognitive dysmetria model, disruptions in connectivity of the prefrontal lobe, the thalamus nuclei, and the cerebellum might be associated with difficulties in regulating, processing, coordinating, and responding to information among patients with schizophrenia. In line with the theory, Bernard and coworkers [28] reported that increased postural sway in the youth at ultra-high risk for psychosis was associated with decreased cerebello-cortical connectivity, as detected by functional connectivity magnetic resonance imaging (fcMRI). They suggested that impairments in sensory integration potentially contributed to poor postural stability, which may partially explain the results of this study.

### Relationships of Postural Stability and Clinical Characteristics

In our study, postural stability of patients with schizophrenia did not correlate significantly with clinical characteristics, including the illness duration, general psychiatric symptoms, negative psychiatric symptoms, involuntary movements, and the CPZE level of antipsychotics. This is in accordance with the results of previous studies [1,4, 6, 14, 27]. However, the influence of antipsychotics on balance control cannot be precluded in this study. Antipsychotics-induced movement disorders, including akathisia and parkinsonism, are prevalent in patients with schizophrenia [42], which interfere with movement performance including postural control. Atypical antipsychotics, such as quetiapine [2] and risperidone [43–45], as well as typical antipsychotics, such as haloperidol [45], were reported to be related to increased postural sway in healthy youth or the elderly. Koreki and coworkers [2] explored the relationships between postural sway of patients with schizophrenia and antipsychotics, revealing no effects of CPZE levels of antipsychotics on the range of postural sway. However, when they divided antipsychotics into high- and low-dose groups as well as different types, they found that postural sway range was greater for patients receiving antipsychotics with a CPZE of 10 mg/d or greater as well as for those receiving quetipine [2]. Therefore, non-specified total CPZE dose may not be a sensitive indicator of the effect of antipsychotics on postural control. de Groot et al. [45] reviewed the influence of medication on postural control and reported that haloperidol 3 mg, olanzapine 3 mg, and risperidone ≥1 mg increase the postural sway significantly in healthy youth and the elderly. Future studies should identify different dose levels as well as particular types of antipsychotics [39] that affect postural stability of patients with schizophrenia. Additionally, studies have reported that benzodiazepines and antidepressants are related to decreased postural control in healthy youth and the elderly [45, 46]. Since benzodiazepines and antidepressants are commonly prescribed to patients with schizophrenia, their effects on postural control should also be investigated in future studies.

In addition, neurological abnormalities are prevalent in patients with schizophrenia [47–49]. These non-localized impairments in neurological functions are manifested by abnormal neurological soft signs (NSS), including motor dyscoordinations, impairments in sequencing, compromised sensory integrations, and cognitive deficits [5, 48, 50–54], which indicate brain abnormality of patients with schizophrenia, such as dysfunctions in the motor-integrating system [55]. Both spontaneous and antipsychotic-induced movement disorders, including involuntary movements, parkinsonism, and tardive dyskinesia have been reported to be prevalent in patients with schizophrenia [29, 30, 56], However, only one posturography study has examined the effects of parkinsonism on postural stability and revealed no relationships [4]. Although the results of the current study indicated no correlation between involuntary movements and postural stability, no conclusion could be drawn currently due to limited number of studies reported. Therefore, further studies should explore the relationship between postural stability and movement disorders in patients with schizophrenia.

Fujino and Imura reviewed nine studies that investigated the relationships between clinical characteristics and postural sway, as determined by posturography in psychotic patients [39]. They concluded that variations in postural indices and conditions resulted in inconsistent findings and precluded comparisons across studies. They also suggested that equality in postural control assessments should be assured to be able to compare the results [39]. Nevertheless, given that postural control is a complex function, the results from different postural variables and testing conditions reflect diverse aspects of postural function and altogether advance our knowledge of the mechanisms of postural control. Homogeneity of conditions and postural variables, as proposed by Fujino and Imura [39], would prevent a comprehensive scrutiny of postural problems of patients with psychotics, including schizophrenia. As of today, only a limited number of studies have used posturography to assess postural stability in patients with schizophrenia; hence, future work on the relationships between various indices for postural stability and clinical characteristics might reveal information that is more specific.

### Clinical Implications

In this study, we included chronic patients with schizophrenia who had limited psychotic symptoms and relatively good physical and cognitive functions. Nevertheless, these patients still demonstrated significant impairments in postural stability. Postural dysfunctions as well as motor abnormalities are often regarded as consequences of antipsychotic treatments [3, 40]; however, spontaneous involuntary movements have also been found in antipsychotic naïve patients [30, 56]. The results of the current study highlight the issue of postural control problems in patients with schizophrenia; therefore, evaluation and training programs should be included in regular intervention protocols to address postural dysfunction.

### Limitations and Future Directions

Due to a relatively small sample size, the results need to be interpreted with caution. Additionally, since chronic patients with few psychotic symptoms were recruited from day-care in this study, the results may not be applicable to patients with schizophrenia in the acute state. In addition, the use of the locally revised version of BPRS to measure psychiatric symptoms may prevent the comparison of the severity of psychiatric symptoms of the patients with schizophrenia across studies. Furthermore, the current study did not explore the effects of benzodiazepines and antidepressants on postural sway besides antipsychotics, leaving the potential influence of both benzodiazepines and antidepressants on postural stability undetermined.

In this study, the sensory organization test was used to scrutinize postural control function of patients with schizophrenia by testing their ability to maintain a static standing posture when exposed to incongruent sensory information. Future studies may also examine postural control in self-initiated, goal-directed movements that feature daily activities, or even directly examine postural stability in performing functional activities. These studies should provide further information on postural control in patients with schizophrenia.
